# Masculinities on the Continuum of Structural Violence: The Case of Mexico’s Homicide Epidemic

**DOI:** 10.1093/sp/jxx010

**Published:** 2017-09-14

**Authors:** Jennie B. Gamlin, Sarah J. Hawkes

**Affiliations:** Institute for Global Health, University College London, 30 Guilford Street, London WC1N 1EH, UK

## Abstract

Through the theoretical lens of a “violence continuum” we explore how, in many of the most marginalized areas of Mexico, global and regional historical and contemporary structures have shaped and constrained men’s ability to achieve the hegemonic masculinity of neoliberal Mexico. An analysis of statistics and local research studies on male homicide is used to understand how impoverishment and extreme inequality can undermine men’s capacity to access a dignified standard of living and exercise their masculinity, in the process of which many draw on interpersonal violence as a resource for respect and manhood.

## Introduction

At the peak of Mexico’s counterinsurgency war, anthropologists Aubry and Inda wrote a short article for the Mexican *La Jornada* daily titled *Quiénes son los “paramilitares”?* (Who are the “paramilitaries”?). Paramilitary groups were widely seen as responsible for the ongoing violence against Zapatista communities, and in particular the massacre of forty-seven Mayan Indians living in the community of Acteal. Themselves indigenous Mayans from nearby communities, mostly affiliated to the ruling Revolutionary Institutional Party (PRI), paramilitary groups such as “Paz y Justicia” operated as counterinsurgency militia, spreading terror, death, and conflict. In their article, Aubry and Inda provided what amounts to a social epidemiology of the paramilitary condition:

[T]he agrarian inertia combined with demographic growth gives neither land nor work, at least not agricultural, to young men who are old enough to acquire a piece of ejidal (communally owned) land. Those who are married, like their parents, have been vagrants in search of work, surviving on miracles or thefts from other peoples’ land. Forced to live like delinquents, not only do they lack a means of subsistence but they have no reason to attend community assemblies and so become excluded from decision making processes in the same ejidos in which they have become pariahs. First conclusion: these criminals are products of the system and their economic, agrarian and employment options.

The authors go on to describe how “paramilitarization offers them a solution and prestige,” a solution because the “war taxes” and thefts of animals and crops provide income, and prestige because the guns they are provided with confer a “power and status that neither they nor their landless parents have ever experienced” (Aubry and Inda 1997).

Like anthropologist Lewis (2006[1959], 2012[1961]), and more recently Philippe Bourgois (1996, 2004) and Hume (2004), Aubry and Inda directly link male expressions of violence to a political economy which has undermined men’s ability to provide for their families, particularly among the most marginalized members of society.

In this paper, we expand on the idea that gender systems and the “doing” of gender are intricately linked to the *global* political economy, and propose that the identities of many Mexican men who belong to criminal gangs are bound up with a “disobedient” form of masculinity that is driven by structural conditions of inequality.

### A Political Economy Understanding of Masculinity

Drawing on theoretical work on the *doing* of masculinity (Connell 2005; Connell and Messerschmidt 2005; Donaldson 1993; Gutmann 1996; West and Zimmerman 1987), empirically grounded theory on the interactive nature of masculine identity and violence (Baird 2012; Bourgois 1996; Messerschmidt 2000), and structural theories of self-realization and agency (Bourdieu 2001; Bourgois 1996; Valencia Triana 2012), we explore how inequality and the corresponding social and economic subordination that it generates has impacted on gendered roles, expectations, and behaviors, and argue that these are leading to increased violence among men from the most deprived sector of society, generating a gendered health effect (Hawkes and Buse 2013) in the form of exceptionally high male death from homicide.

This discussion sits alongside a growing body of research on gang-related violence and identity in Latin America, and among Hispanic populations in the USA (Alcalde 2011; Azaola 2012; Baird 2012; Bourgois 1996, 2004; Hume 2004; Jones and Rodgers 2009; Pereya 2012; Reguillo 2012). With specific reference to homicide that occurs around and between drug cartels and organized crime in Mexico, we argue that structural violence, in the form of poverty, inequality, and deprivation, has restructured traditional male identity in reference to the hegemonic masculine identities embedded in national and global political economic orders. The neoliberal economic model that Mexico has followed for the past three decades has led to a deterioration of essential services and economic conditions, resulting in the exclusion of large sectors of the population from education and employment opportunities (Reguillo 2012). The more than seven million young Mexicans between the ages of 16 and 29 (Solano 2015) who comprise this “industrial reserve army” (Harvey and Reed 1996, 479), referred to derogatively as *ni-nis* since they are neither employed nor in education, have become the *waste products* of economic restructuring, left with little option but to make a living in the already saturated informal sector (Arizpe 2015), an increasing proportion of which is controlled by organized crime.

The specific form of neoliberal capitalism, referred to by Valencia Triana (2012) as “Slasher Capitalism,” that has become endemic in Latin America is a particularly cruel and unequal form of the global neoliberal model (Arizpe 2015; Fazio 2016; Kraniauskas 2012). It has become an “economy of violence” (Suchland 2015) with aberrational consequences for human behavior and social relationships. Drawing on gendered theories of violence and structural violence (Connell 2012; Mies 1998; Segato 2010; Suchland 2015; Valencia Triana 2012), we take a historical view of masculinity and political economy in Mexico to describe how it has become a breeding ground for a particularly violent male identity that in other parts of Latin America is generating homicide rates of above 100/100,000 (InSight Crime 2016).

### A Political Economy Perspective of Homicide as a Gendered Health Effect

By the end of 2015 a total of 32,791 homicides had been recorded in Mexico, marginally up on the 2014 figure of 32,631 (Secretaria Ejecutivo 2016). Although the rate declined by around 4 percent between 2012 and 2015, interpersonal violence is still the commonest cause of death among men aged 15–49 years in the country. It is estimated that 88 percent of all homicide victims are men, and 90 percent of these men are murdered by other men (Menéndez 2009), with rates highest among men in their twenties and thirties (Gamlin 2015).

There is much discussion about Mexico’s murder rate in the context of cartel violence (Guerrero Guttierez 2011; Rios and Shirk 2011). The official position is that these are mostly murders that take place between drug cartels and security forces, emphasizing a linear relationship between the increase in homicide, and growth of narcotics-related organized crime (Norzagaray Lopez 2010; Pereya 2012). The authors of this paper are under no illusions about the fact that homicide and organized crime have increased significantly over the past decade. There is clearly a need to address this problem on the ground using security forces and intelligence, but problematized in this way, the “solution” has been to address this violence with further violence at the hands of security forces. Throughout this circle of violence, drugs-related activity and trade has essentially become a cover for what is a crisis of human rights and social structural failure, as the Mexican state neglects to protect and provide for its population. This reductionism and invisibilization of the deaths of tens of thousands of marginalized young men also avoids the urgency of analyzing the social and historical processes that have led up to this and working towards structurally focused solutions, a point taken up more broadly by Moser and McIlwaine (2014) in their discussion of twenty-first century urban conflict and violence.

Our position is that the current pattern of male mortality from homicide in Mexico is a *gendered health effect*, a result of the process through which men and women have been drawn into history and the manner in which they live out and define their gender identity in relation to other people: in intimate relationships, within the family, peer groups, work, and society. Simply being male is a risk factor for experiencing a violent death, as it is for dying from other activities related to gendered risk-taking behavior and institutional neglect of men’s own wellbeing—smoking tobacco, alcohol consumption, and driving-based employment (Connell 2012; De Keijzer 2001; Hawkes and Buse 2013; Lim, Vos, and Flaxman 2012). Globally, men are significantly more likely than women to suffer morbidity and mortality associated with interpersonal violence, which is “peace-time” violence, as opposed to that associated with conflicts and wars (Hawkes and Buse 2013). These practices through which we *do gender* (West and Zimmerman 1987) also form part of a social structure, and as such are subject to constraints, approvals, and disapprovals, based on dynamic cultural ideas about how men and women should behave; but also in the form of strategies for survival and practices that are aimed at mitigating the hurtful impact of structural inequalities.

Men in Mexico have 6.5 fewer years of life expectancy than women—a finding that cannot be explained by biological sex alone. The Institute for Health Metrics and Evaluation (which produces the agenda-setting Global Burden of Disease data) confirms that life expectancy for men and women globally should be equal since “there is no reason that society should have lower aspirations for health for males than females” (Murray et al. 2012). In Mexico, as elsewhere, it is likely that male/female life expectancy gaps are driven more by gender than sex. For example, in the age group 15–49 years, Mexican men have a death rate from violence that is five times higher than women’s (19 percent of all male deaths in this age group, compared to 4 percent of female deaths) (IHME 2015). Gender is also a key determinant of Disability Adjusted Life Years (DALYs) with interpersonal violence coming in as the leading cause of DALYs for men, while it does not feature in the top twenty female DALYs. Similarly road injuries are in third place for men, but only twelfth for women. In contrast, major depressive disorders are the third highest ranking DALY for females and only fourteenth for men (IHME 2013).

While this excess male mortality and morbidity is a product of masculine agency and identity, it is also a form of structural violence—indirect forms of harm exerted by social structures and institutions—enforced on both national and global levels, which carries negative consequences for the health and well-being of men and women. While the data may show the excess morbidity and mortality suffered by men in Mexico, the root causes of much of the violence suffered by men and women in Mexico is the same: the inequality inherent in global economic institutions, which has become normalized in economic relationships (Suchland 2015, 12).

We challenge this normalizing effect by discussing how these global social, economic, and gender structures have shaped manhood and masculinity in Mexico. Our argument is situated within the social and historical context of machismo in Mexico, where we explore how culturally defined male identities that did not depend on accumulating material wealth and symbols of status have been replaced by the hegemonic masculinities that have emerged under neoliberal capitalism. Evidence of the current context of violence in Mexico is drawn from locally generated research and national government statistics on violence, homicide rates, and organized crime. We interrogate this evidence from the theoretical position of structural violence to explain how changes to socioeconomic and gender structures have redefined men’s identity in relation to new forms of hegemonic masculinity.

For the purpose of this paper, we define hegemonic masculinity as a “culturally idealized form of masculine character,” although this may not be the “usual” form of masculinity (Donaldson 1993, 646–7).

To date, the debate about how gender and structural violence intersect has centered largely on the forms of homicide that impact upon women, such as intimate partner violence (Radford and Russell 1992; Wright 2011). The term femicide—*feminicidio* in Spanish—is widely used to describe misogynous murders of women by men, viewed as a continuation of the sexual violence generated by imbalances of power between men and women in economic, social, and political spheres (Monarrez 2002, 283). We draw on the rich body of *feminicidio* literature to advance an argument that the violent “doing” of masculinities is representative of both direct and structural violence but it is also *symbolic*. Drawing on Bourdieu’s theory of masculine domination, which describes how structures of domination become violent through their constriction of agency, we reframe an interpretation of symbolic violence, empirically documented though not explicitly theorized in the biographical–anthropological works of Lewis (2006[1959], 2012[1961]), with reference to the doing of marginalized masculine identities as a form of gendered social practice that is violent to both victims and perpetrators.

## The Historical Continuum of Structural and Interpersonal Violence

In order to understand the roots of today’s homicide epidemic, we need to review “how the violence of economic inequality, unemployment, and precarious employment has translated into chains of violence, from violence against women to the most dehumanized forms of criminal violence” (Arizpe 2015). Mexico has a history of violence between the powerful and against the powerless, well documented in studies of pre-colonial civilizations, which intensified during the conquest and under colonial rule, and carried through into the war of independence, the revolution, and beyond (Krauze 2012). In 1930, the Mexican Ministry of Health detailed a rate of seventy-seven homicide deaths per 100,000 of population, gradually declining to 17.5 in the 1970s and 1980s (Azaola 2012), and to 8.0 at its lowest point in 2006 (Davila-Cervantes and Pardo-Montaño 2013). This is a history and contemporary context shared with many other Latin American counties—see for example Menéndez’s (2002) account of the historical trajectory of post-colonial violence in Argentina, Segato’s (2010) account of the role of violence in the formation of Brazilian society, or Hume’s (2004) work on recent political history and criminal violence in El Salvador.

Scheper-Hughes and Bourgois (2004) describe how the varying presentations that have existed throughout history are a *continuum* of forms that give birth to each other, or as Azaola (2012, 18–9) suggests, “there is a continuity between […] political and criminal violence” or, between “the pathologies of power, individual, and collective.” Galtung (1969) used the term *structural* violence to refer to indirect forms, exercised by state and social institutions that are present when avoidable morbidity, constrained potential, or reduced life expectancy are present. Criminal and interpersonal violence in Latin America is increasingly linked to socio economic exclusion and structural factors, making this a form of structurally generated gender violence (Arteaga Botello and Jimena Valdez 2010; Baird 2012; Hume 2004). In her account of the historical development of patriarchy, Mies (1998) describes how the direct violence implicit in capitalist accumulation under slavery transformed into the structural violence of economic coercion, sanctioned politically and economically by states, an account supported by Rita Segato’s recent research with Brazilian indigenous communities (Segato 2010). Mies goes on to argue that this change in production relations from one of master and servant to that of capital and wage labor was itself only possible through the use of large-scale violence in the form of sanctions, deprivations, and punishments. Within this process, extreme forms of exploitation and resulting inequality became the new forms of violence to be seen as the natural order of human society. Research from anthropology, sociology, and psychology demonstrates that aggression happens when the psychological self feels threatened (Chodorow 2002; Donaldson 1993; González Montes 2012; Messerschmidt 2000). We argue that the current form of hegemonic masculinity that is a product of a neoliberal political economy exerts violence against both men and women through threats to masculine identity generated by structures of inequality, which breed further forms of violence.

Forms of structural and indirect violence often begin with “assaults on the personhood, dignity, sense of worth, or value of the victim” (Scheper-Hughes and Bourgois 2004, 1). These everyday forms of violence that are a direct consequence of inequality present themselves as poverty, hunger, social exclusion, and humiliation, and regularly translate into further forms of violence and the eventual lowering of life expectancy, characteristics that Lewis (2006[1959], 2012[1961]) identified within his “culture of poverty” account of marginalized Mexico City households in the 1950s and 1960s. Moreover, structural violence frequently leads to violence in direct interpersonal forms, although the continuum between structural and interpersonal violence is hidden and therefore often overlooked. Separately, Bourgois and Scheper-Hughes highlight this link in their work in the USA and Brazil, illustrating how everyday violence in the forms of poverty, marginalization, loss of livelihoods, and vulnerability are causal factors in domestic gender-based violence against women, and interpersonal conflict among men (Bourgois 2004; Scheper-Hughes 1993). Bourgois directly links late twentieth century restructuring of the global economy with a crisis of working-class patriarchy and gang violence among Latino immigrants in New York to explain why such large numbers of poor men are killing one another. This empirical work with Hispanic populations reinforces the notion of Parsons (1954) that the occupational system is the most important process through which individuals—in this case men—achieve their status, with an absence of opportunities acting as a blockage to manhood (Bourgois 2003).

In summary, the link between global economic restructuring and violence is visibilized when we focus on how poverty, marginalization, and consequent male unemployment directly impact on men’s sense of worth and dignity, which then acts as a trigger for different forms of violence.

Research with men in Mexico has also shown a clear pattern linking structural and interpersonal violence: as opportunities for men to make a living are increasingly restricted, domestic violence increases (Torres Falcon 2004; González Montes 2012). In particular, a recent analysis of homicidal violence against women provides evidence for the conclusion that one determinant of these femicides is the collapse of hegemonic models of femininity and masculinity (Arteaga Botello and Jimena Valdéz 2010). Recent anthropological research also points to the role of historical events and structures in interpersonal violence (Segato 2010), and to the impact of living in psychosocially violent family contexts characterized by extreme poverty, brutality, and social exclusion on the use of extreme forms of interpersonal violence (Arizpe 2015). These various forms of violence feed each other in a non-linear manner where it becomes impossible to interpret any one in isolation from the other. To understand the nature and magnitude of Mexico’s current epidemic of male violence and homicide, we must uncover how the structural violence inherent in social structures of gender and socioeconomic inequalities has contrived to generate the specific forms of direct violence that are operating today.

## A Political Economy of Masculinities

### *Machismo* and Mexican Masculinities before Neoliberalism

*Machismo* is a concept used to refer to a stereotypical form of masculinity, although there are multiple and more nuanced characterizations of a “macho” Mexican, as has been demonstrated by numerous anthropological and sociological studies (Figueroa and Jiménez 2006; Gutmann 1996; Lewis 2012[1961]). In Mexican Spanish, the concept has its origins in a cultural context that represented the hegemonic masculinity of a pre-globalized era. This was the Mexico of *campesinos* (peasant-farmers), in a time when masculinity was not measured in material or economic terms. Machos were family men of honor and courage, poor but brave, fierce but trustworthy, “we have nothing but we are real men” (Monsiváis 2013, 53).

Deeply interwoven with the idea of nationalism, masculinity became a cornerstone in the construction of a Mexican national identity (Chant and Craske 2007; Gutmann 2007; Valencia Triana 2012), enshrined in the Golden Era of Mexican cinema (late 1930s to early 1950s) and celebrated in folk and country music: “Your men are macho and dutiful, brave, fierce, and reliable, they accept no rivals in the things about love” (Monsiváis 2013, 55). Thus, “Mexico came to mean machismo, and machismo to mean Mexico” (Gutmann 2007, 224). A functional hegemonic masculinity stabilizes the structures of dominance in the gender order and is naturalized in the form of the hero—thus its representation in films, ballads, westerns, and thrillers (Donaldson 1993). While built on a highly unequal gender structure, Mexico’s macho identity was a hegemonic masculinity that ordinary people could access. It was also a moral identity, embodying the values of the post-revolutionary Mexican republic. At a time when the possession of wealth and fortune were not defining factors in the attainment of masculinity, Mexican *machismo* was both popular and hegemonic, while its marriage with nationalism naturalized the patriarchal structure of society. This was also a time when there was pride in being poor, and the stigma of poverty had yet to be felt to undermine the doing of masculinity, making it a hegemonic masculinity accessible to all (Arizpe 2015).

### Economic Change and Gender Identity

Firmly established in the *doing* of machismo is a man’s responsibility for supporting his family, and as Mexico transitioned from an agricultural to an industrial economy, financed by the high rates of growth that protectionism was bringing, the state sector was to some extent able to provide new forms of employment though investment in health and social sectors. Although rapid urbanization in the second half of the twentieth century undermined the classical notion of peasant masculinity, the growing economy gave relative stability to the gender order and men’s position of power as the provider and head of the family. These transitional decades after the World War II saw massive rural–urban migrations within Mexico alongside progressive undermining of traditional social structures. These dynamics are documented by Lewis who alludes to the impact of political economy on kinship structures and in particular on male lives. Although Lewis’ work has been much criticized, in particular, the suggestion that he is blaming the poor for their own hardship, we share the perspective of Harvey and Reed (1996) and Bourgois (1996, 2001) that Lewis’ work is in fact a seminal account of structural violence. More recent discussions of gender violence in Mexico have reiterated this position (Chant and Craske 2007, 316–8; Arteaga Botello and Jimena Valdez 2010)

As Gutmann (2007, 238) points out, “identities only make sense in relation to other identities,” and the negative stereotyping of Mexican machos by its northern neighbors was later replicated within the middle and upper social classes. Although in the latter part of the twentieth century machismo became denigrated as a lower class identity, the patriarchal practices and gender universals of a sexual division of labor remained strongly entrenched. Compounded by the Structural Adjustment Programs imposed by international financial institutions in the 1980s under the Washington Consensus, the stigma associated with being poor emerged as urbanization left increasing numbers of *campesino* families abandoning rural poverty to seek a precarious existence in crowded urban settlements (Lewis 2012[1961]). These changes were to contribute to the stratification of Mexican masculinities, with machismo attached to the ideas of lower classes (Monsivaís 2013) giving way to a new globalized hegemonic masculinity underscored by notions of material wealth and power.

### Structural Violence and Hegemonic Masculinities

In his ethnography of poverty, Lewis noted that “the Mexican capacity for suffering has its limits … and we should expect that sooner or later there will be social disorders” (2012[1961], 51). Within the social structure of gender, the principal organizing factor is a gender-based allocation of tasks that has become embedded in social practice, forming the basis of social structures of domination and inscribing durable effects on the body, mind, and dispositions that are reproduced through the *habitus* (Bourdieu 2001). Central to Bourdieu’s interpretation of gender as a structure of domination is the tension between forces of constraint, and submission or voluntary actions of individuals.

Bourdieu explains how social structures constrain agency while at the same time symbolic forms of violence are enacted to deal with this. This system of masculine domination has become deeply ingrained, exerting “durable effects on women,” that is to say, “dispositions spontaneously attuned to the order which it imposes on them” (Bourdieu 2001, 38). These dispositions, actions, and subconscious forms of doing gender are subject to conscious acts of social endorsement and chastisement, processes through which men and women learn how to behave in a manner that is appropriate for their gender, at once agentic and a matter of social structuration. It is from this position that Bourdieu (2001, 38–9) proposes the concept of symbolic violence as a primary form of oppression, referring to the manner in which the dominated, “tacitly accept the limits imposed” and “unwillingly contribute to their own domination” as an act of agency and through reproduction of the system. The effect of this symbolic domination is violent because it takes the form of emotional and psychological harm including humiliation, anxiety, shame, and guilt. This invisible form of violence, existing primarily at the household and individual level, is a self-generating form of violence that gives birth to further forms [see Bourdieu (2001), on symbolic violence, 33–42].

The location of symbolic violence is well evidenced when approached from the perspective of masculinities. Bourdieu (2001, 35) uses examples of objective forms of domination to illustrate how symbolic violence plays out in women’s lives and is reproduced through the habitus, a position that has been applied to work on violence and masculinities (Baird 2012). We further complicate this theory by exploring how the same structures of domination also constrain masculine dispositions and bring symbolic violence upon men in Mexico. Within Bourdieu’s dispositional theory of practices, while women adopt behaviors attuned to their position of being dominated, men practice domination publicly and within the family. They are socially required to adhere to a specific set of gender practices that are structurally determined—the doing of masculine identity through agentic behavior as a learned set of practices within their habitus. The symbolic power of a gender structure, which consigns women to separate spaces and generates submissive attitudes, obliges men to reinforce their superior position by fulfilling their primary masculine obligation of providing for and protecting the family. Men, therefore, are subject to symbolic forms of violence though the self-harm inherent in achieving masculine identity: the loss of dignity and sense of inadequacy, failure, and humiliation at their inability to conform to a hegemonic ideal. The violence of patriarchal capitalism over men is compounded through the symbolic power of hegemonic masculinity.

In his account of the construction of masculinity among Colombian gang members, Baird (2012, 182) draws on the concept of habitus, which he suggests “disposes youths to make their own transition into a culturally valued, and thus common and reproduced gendered self; expressing agency and dynamism in doing so”. While habitus operates in the social reproduction of violence, this is part of a continuum which includes the stigma and humiliation of emasculation, the social position of dis-dignity that erupts from the inability to achieve a desired social and gender status, and the extreme violence generated “in search of respect”.

There is an inseparability of the various forms of violence that we trace through the relationship along a continuum and through the ongoing construction and redefinition of masculinities. The diverse manifestations of violence are born of different causal factors, but are interrelated not only through definitional continuums but through the structures of masculinity and gender within patriarchal capitalism, which also legitimize violence within interpersonal relationships. Some of these may be violences “produced in the structures [and] habituses” that are formed by class, race, and gender inequalities (Scheper-Hughes and Bourgois 2004, 19). These structural violences share a common denominator of the ability to cause harm to human integrity, whilst placing the same people in a position of increased vulnerability to physical and life-threatening forms of violence. Within the context of neoliberalism, men’s ability to generate income for their families and to fulfill the gender-specific life expectations defined in relation to hegemonic forms of masculinity is undermined by structural forms of violence generated at a global level. Concretely, as structural adjustment policies took hold in the 1980s, men’s ability to earn sufficient income to maintain their family diminished and women began to enter the labor market in ever larger numbers, resulting in changing gender dynamics that essentially undermined the bread-winner model of masculinity (Arizpe 2015; Monsiváis 2013). This gendered labor market dynamic is widely reported in relation to masculinity and violence (Arteaga Botello and Jimena Valdez 2010; Chant and Craske 2007). The response has been to seek alternative means of generating income and defining a masculine self. A similar scenario is described by Bourgois in “In search of masculinity” (1996, 413) with reference to Puerto Rican gang members: “Unable to provide economically for their conjugal unit they lose the material legitimation for demanding autocratic respect and domineering control over their wives and children.” The same principle of a disrupted gendered identity dynamic has been signaled widely in research on interpersonal violence that men inflict on women as well as studies of femicide (Arteaga Botello and Jimena Valdez 2010; Chant and Craske 2007; González Montes 2012).

In this “post-socialist” era, male identity, now intrinsically linked to the ability to generate income, faces challenges in the form of humiliation, shame, trauma, failure, or lacking recognition, symbolic forms of violence that can generate an aggressive response (Chodorow 2002; Turan et al. 2016). Connell (2005) uses the term “protest masculinity” to describe a masculinity that lacks the resources to reproduce the hegemonic model and so seeks alternative, often violent, sexual, or illicit means of imitating the power that sustains the hegemonic model. The social disobedience required to construct this power gave its name to the idea of *disobedient masculinities* (Valencia Triana 2012). Where these are embedded in a context of extreme poverty, this has become “marginalized masculinity” (Connell 2005). Underlying all of these concepts is the process of “grappling with a situation and constructing ways of living within it [that] is central to the making of gender,” in a manner that is in some way “complicit with the collective project of patriarchy” (Connell 2005, 114), while this hegemonic project remains beyond reach.

## Political Economy of Poverty and Masculinity

### From Macho to Marginalized in Late Twentieth Century Capitalism

In the 1970s, Mexico began its path to neoliberalism and the changes that ultimately led to today’s social dystopia (Monsiváis 2013; Valencia Triana 2012). The decades of structural transitions that were a consequence of the global turn towards neoliberalism impacted the *doing* of Mexican machismo. As the motor for social change, these economic changes brought a decline in purchasing power of the male wage and structural changes to the gender division of labor.

After a period of relative stability in the 1970s and 1980s, excess male mortality began its ascendance again in Mexico mostly driven by interpersonal violence (González Pérez et al. 2012). Between 2006 and 2011, the national homicide rate more than doubled, together with the rapid increase in narcotics-related activity and organized crime, leading one epidemiological analysis to suggest that in the first ten years of the new millennium violence has led to a “stagnation” of life expectancy, implying a decline in real terms (Canudas, Garcia and Echarri 2015). This increase in violence led to the conclusion that the cause of these deaths among young men was involvement in criminal activities (Bergman 2012; Dávila-Cervantes and Pardo-Montaño 2013; Menéndez 2009). At the same time, a large body of work analyzed the rise in female homicides in terms of the historical, social, and structural determinants of these murders as “femicide” (González Rodríguez 2002; Wright 2011). The well-documented rise in femicides over the past two decades speaks both to changing masculinities and femininities that are the result of structural gender dynamics, and a generalized decomposition of the state, reinforced by criminality and its infiltration within the government. As Monarrez puts it, “The death of women is an expression of gender oppression, the inequality of relationships between masculine and feminine, between domination, terror, social extermination, patriarchal hegemony, social class and impunity” (2002, 281–2).

These violent deaths have become an ongoing human rights concern. A study of femicides in Mexico that analyses case-by-case data on murders concludes that these are a response to the “collapse of hegemonic versions of masculinity and femininity” in contexts of poverty (Arteaga Botello and Jimena Valdez 2010, 6). We argue that the gendered analysis used to explain femicide is equally relevant to understanding male-on-male homicide.

So far, the largest burden of mortality from homicide occurs among young men—a phenomenon predominantly studied as a crime statistic and national security issue, with a lack of concomitant gendered analysis. In the project of neoliberalism “masculinity is legitimated though purchasing power” (Valencia Triana 2012, 83), but in a country where 52 percent of the population live below the poverty line, the majority of Mexican men can only ever dream of acquiring symbols of consumerist success (The World Bank 2017; Chant and Craske 2007). The hegemonic masculinity of Mexico closely mirrors dominant global models of patriarchy, which are reinforced by globalized cultural forces (advertising, commercial pressures, cinema, literature, etc.). To reflect back on the “hero model,” hegemonic masculinity is personified by actors of romantic heroes such as Richard Gere and Harrison Ford, sports heroes such as David Beckham, or the powerful political heroism played out by leaders such as Barack Obama.

The specific form of neoliberal capitalism that operates in Mexico is structured to ensure the growth of extreme economic inequality largely through exploitation in “slasher” capitalism: “exacerbated neoliberalism, globalization, the binary construct of gender as political performance, and the creation of capitalistic subjectivities” (Valencia Triana 2012, 83). In the absence of an effective taxation or welfare system to redistribute wealth, and with an abundance of labor to suppress wages, neoliberal capitalism in Mexico has taken on a particularly violent form, a “savage capitalism” where the extreme exploitation of both consumer and worker underpin gross inequality (Arizpe 2015; Fazio 2016; Kraniauskas 2012). According to Suchland (2015, 7), these are “Economies of Violence,” “post-socialist” nations—including countries in Africa, Asia, and Latin America—in which “the state socialist projects have collapsed” and forms of violence thrive that are part of the national and global political economy as well as racial and patriarchal norms. The naturalization of this form of structural violence is not recent. Inequality has been knitted into the process of global capital accumulation to such an extent that it appears to be part of the natural order of things. Even in its extreme forms, inequality has come to be seen as a justifiable and a natural attribute of progress and development. In this late period of neoliberal capitalism, the idea that the concentration of wealth in the hands of a few is actually good for humanity because it constitutes the motor for growth has become an accepted economic doctrine (Boix 2009; Piketty 2014).

The Mexican state struggles to manage even a marginal redistribution of wealth through the taxation system. The political economy is so riddled with corruption and clientelism that wealth only circulates within a thin slice of the population. Such a system can only exist and operate with governmental complicity, and so it is buffered by an almost absolute state of lawlessness (Arteaga Botello and Jimena Valdez 2010; Gamlin 2015; Tuckman 2012). On a national level, given as a percentage of crimes that lead to an “effective” conviction, impunity runs at almost 100 percent: in 2007, the official figure stood at 98.76 percent, an increase of almost 9 percent on the 1998 index (SIISP 2013). Corruption is a top-down phenomena and in Mexico high-ranking political figures, including successive presidents, have led by example. This situation is not unique to Mexico and we surmise that similarly failed states underpin the epidemics of violence against both men and women in Central America and Venezuela (Baird 2012; Hume 2004).

This specific form of neoliberal capitalism has been prosperous in large part thanks to its position on the fringes of legality, buffered by a parallel economy of organized crime (Fazio 2016; Solís González 2013). The flip side of Mexico’s neoliberal economic growth has been the siphoning away of resources that once supported national health systems, universal primary education, employment stability, and pensions in the state sector and support for the tens of millions of peasant farmers through subsidies and protectionism. This move towards neoliberalism came at a time when the largest population cohort was reaching working age, and institutional provision, particularly of further education but also across the range of health and social provision, failed to keep up with demand. In 2006, a year after the 18–25 population reached its historic maximum peak^1^, 22.1 percent of Mexicans between the ages of 12 and 29 were without access to places in further education or employment (Reguillo 2012). The state’s failure to meet the capabilities and enhance capacity for such a huge sector of society (Sen 1999) expanded what Reguillo (2012, 39–40) has termed the “pirate imaginary”: “the inclination to accept and even value extra-legal practices that range from drug consumption to taking justice into their own hands.” In 2007, men under the age of 25 participated in 67 percent of all homicides related to organized crime, and 49 percent of all deaths from homicide are young men of the same age (Reguillo 2012).

While the figures presented above refer to Mexico as a whole, the manner in which this context of marginalization is played out in men’s lives is politically and spatially differentiated. In her analysis of femicide in the border city of Ciudad Juárez, Monarrez (2002, 283) explains how “the degree to which social groups tolerate structural changes is evidenced in the levels of social violence,” a violence that appears to be more common in urban and slum areas of developing country cities (Chant and McIlwaine 2016, chapter 6; Moser and McIlwaine 2014). Lewis and Guttman base their studies of marginalized masculinities on the outskirts of Mexico City during a time of rapid rural–urban migration. While undoubtedly the gendered interaction between violence and structural economic change is occurring simultaneously, it has not necessarily led to the same consequences throughout the country. In recent years, male–male homicides have been concentrated in states such as Michoacán, Veracruz, and Guerrero, where the conditions of poverty combine with a fertile geography for drug production. A comparison of regional homicide statistics is beyond the scope of this paper, but we hypothesize that these variations in murder rates are associated with a complex interplay of political economy and masculinity with geographical, cultural, and historical factors, a dynamism that would also seem to be reflected in the temporality of homicide rates.

### Gender, Structural Violence, and Homicide

Reguillo’s research provides compelling evidence of the agency and structures that have delivered tens of thousands of young people into the violence of organized crime. The Mexican state and society send its youth contradictory messages. On the one hand, a culture of hyper-consumerism dictates that individual, family, and social identity are achieved through ownership of purchasable status symbols. On the other hand, with more than half of the population living in poverty, the opportunities to access resources are extremely limited for the majority of Mexican society. In his analysis of hegemonic masculinity, Donaldson (1993, 643) refers to structures of oppression as motors for social changes that impact upon the gender systems. Citing Hochschild, he describes “the decline in purchasing power of the male wage, the decline in number and proportion of ‘male’ skilled and unskilled jobs, and the rise in ‘female’ jobs in the growing services sector” as key players in the gender order. These are only some of the social changes that have left the current hegemonic masculinity beyond the reach of large swathes of Mexican youth.

Although the structural factors behind the current epidemic of homicides linked to organized crime continue to be ignored in official discourse, common causal elements have been identified as (i) a weak state and institutions (corruption, impunity, generalized lack of support, and trust in government), (ii) deficient social policy (extreme levels of poverty, marginalization, deteriorating health and education provision), and (iii) a historical propensity to violence throughout swathes of Latin America (Azaola 2012; Bergman 2012; Mercille 2011). Agamben’s concept of a “State of Exception” is frequently cited in reference to current social and political context in Mexico and Latin America (Fazio 2016; Reguillo 2012; Valencia Triana 2012), taking discussion beyond the idea of a “crisis” of governability, an accepted condition in some of the most violent states of the republic^2^, into the realms of state failure.

When the state is weak, cartels move in to provide employment where there are few other options. Fields left empty by farmers who could no longer afford to produce in the “free market,” have become fertile ground for the production of drugs (Arizpe 2015; Fazio 2016). Not only have drug cartels infiltrated the state, in many places they have assumed the role and responsibilities of the state (Solís González 2013). These factors make a life in organized crime not simply a last resort, but an *opportunity*. Crime has become a life choice for those young men disenchanted with the set of political, social, and employment options, the social dystopia that can only offer a continued life in degrading informal work and poverty. To these young unemployed men, organized crime in fact presents itself as a force able to offer wealth or access to a minimum of wellbeing and most importantly, “a sense of belonging, of future, of solution” (Reguillo 2012, 41) and a means of achieving real manhood.

Termed “necro-empowerment,” Valencia Triana describes how violence has become a tool for rapid wealth generation where legitimate means of acquiring wealth are out of reach. In addition to the opportunity for making money, the violence that is part of these forms of criminality also responds to the masculine demand for power and prestige, suggesting that violence in some way offers a rite of passage to manhood. The simple possession of a fire-arm is itself a tool for men to assert their masculinity, particularly within a context of depravity (Carlson 2015; Gutmann 2007). Within the business of organized crime, guns are mostly used as a threat or to maim or kill but they also serve the more basic masculine requirement of protection, giving them a symbolic meaning as well as serving as a resource for achieving power.

## Conclusion

We began by asking “Who are the *narcotraficantes* who are dying in this epidemic of violence?” We end by concluding that these are young men who had no dignified employment opportunities, a poor-quality education that ill prepared them to earn a living, who have been failed by the global and local structures that should be there to protect and guarantee their human rights. They are the brothers, sons, fathers, husbands, and uncles of families who have struggled to make a living and have been defrauded by the state. Aubry noted that criminalization offers “power and status that neither they nor their poor parents have ever experienced.” In addition, among marginalized sectors of Mexico, the violent criminal activity fuelling much of the epidemic of homicide is a result of a disobedient masculine identity driven by the intersection of historical, economic, social, and commercial forces. This intersection is fuelling excess male deaths in Mexico, in part through the “doing” of gender in a context where legal and legitimate resources for achieving masculine respect are inaccessible or unavailable.

It is our position that violence is a natural aspect of masculinity, but as Scheper-Hughes and Bourgois (2004, 22) note, violence is “almost inevitably gendered” and gender “operates throughout all forms of violence.” Nor do we intend to reduce masculinities to the exercise of violence or production of wealth, but rather to illuminate the role of resources in the production of a masculine identity, while recognizing that there are many and diverse ways of “doing” masculinity. The frequent overlapping of violence and masculinity is the reason that violence as a resource to be drawn on in the exercise of power and control has become *naturalized* as a practice in the construction of the latter. Studies in criminology have focused on the relationship between violence and masculinity and found that there was no linear causal relationship, suggesting that men do not necessarily need to be violent per se, but that certain types of masculinity use aggression or violence in the pursuit of hegemony (Connell and Messerschmidt 2005). In his work on the role of sexual violence in making boys into “real men,” Messerschmidt (2000) found that aggression generated rewards for boys who felt they were experiencing a subordinated masculinity in the form of admiration, esteem, and social power. Bourgois (1996) links the struggle for a masculine respect with public physical violence among Puerto Rican gang members in New York, suggesting that these are the only means immediately at their disposal.

Death from homicide is at epidemic levels in Mexico. With one in five deaths of men aged 15–49 years due to interpersonal violence, this proportion is far higher than countries at a similar level of socioeconomic development to Mexico. For example, in the so-called MINT countries (Mexico, Indonesia, Nigeria, and Turkey, a grouping of emerging economies with similar short-term growth prospects), male deaths due to violence are between 1 and 4 percent (IHME 2013). At present, the homicide rate in Mexico has begun to appear low in comparison to other parts of Latin America. In 2015, homicide rates in El Salvador, Venezuela, and Honduras reached colossal highs of 105, 90, and 57 per 100,000 of population respectively (InSight Crime 2016). These crime rates do not alter our analysis of the Mexican case, but it would clearly be of interest to study homicide across the region in order to explore whether a similar link exists between the specific structural drivers of crime that are characteristic of Latin American neoliberal capitalism, and patterns of gender identity and violence. Such an assertion would not be possible without a historical exploration of the three intersecting elements of political economy, masculinity, and violence.

Violence is a structural effect of Mexican neoliberal capitalism that is spatially and temporally variable. In Mexico City and Querétaro state, the potential for violence is abated by strategic governmental control and well-targeted intelligence and security forces, driven by the need to satisfy important national and international capital interests. In contrast, in the states of Tamaulipas and Guerrero where drug production and violence have long-standing cultural and historical roots, Michoacán where poverty and geography have made it prey to marihuana production, or Sinaloa where cartels have taken on a social purpose, organized crime offers desperate men dignity and respect that are otherwise almost impossible to achieve. Across Mexico, but more emphatically in states that for multiple reasons have become heavily involved in organized crime, violence itself has become a resource for masculinity, rewarding the possession and use of weapons and powerful vehicles with admiration, social esteem, power, and wealth as well as the sense of belonging that is central to gang culture.

Modern masculinities are no longer defined by local parameters alone, and the “traditional” has become subordinate and insufficient. The continuum of violence that this structural inequality triggers is not only a violence against women but also against men, both in its symbolic forms and directly through the pursuit of hegemony. Taking agency to mean “an active participation in a world of contested meanings” (MacNay 2000), the manner in which men and women interact with structures of inequality is not passive but happens through the active construction of gender identity within this context of violence. This interaction between political, economic, and gender identity gives continuity to the discussion raised almost fifty years ago by Lewis as he described kinship interactions and adaptations to conditions of adversity.

Much has been written about the need to bring the drugs trade under control, with the belief that as a by-product this will also lead to a reduction in homicide rates. The explosion of violence in Mexico, however, is unlikely to be mitigated through controlling (whether through legalization or other means) the drugs industry alone. Interpersonal violence is tied to a long history of what it means to be a “real man” in Mexico exacerbated by the neoliberal state’s failure in its duty of care towards its citizens. Thus, bringing an end to violence, or at least beginning to see a reduction in its extraordinary levels, will require more than a single (even if challenging) solution. Addressing violence means, at a minimum, recognizing the complex structural, historical, political, social, and economic forces driving the continuum of violence in all its forms.

## Notes

1 According to Reguillo, and based on data collected by the National Youth Survey (2006), in 2005 Mexico’s population of young people reached its peak in absolute numbers, after which numbers began to decline once more.

2 The states of Guerrero and Tamaulipas have each declared themselves ungovernable due largely to the infiltration of organized crime in governmental institutions (http://www.elfinanciero.com.mx/tv/guerrero-caso-iguala.html and http://bakerinstitute.tendenciapp.com/media/files/Research/b0596728/DRUG-pub-HaleTamaulipasFailedState-072611.pdf).
